# Supplementary material for: The circadian clock influences T cell responses to vaccination by regulating dendritic cell antigen processing
Source: Nat Commun. 2022 Dec 5;13:7217. doi: 10.1038/s41467-022-34897-z (PMC9722918; doi:10.1038/s41467-022-34897-z)
Supplement: Supplementary file 1 — Supplementary Information [file 41467_2022_34897_MOESM1_ESM.pdf]

The circadian clock influences T cell responses to vaccination by regulating dendritic cell antigen processing

I. Supplementary table

II. Supplementary figures

I. Supplementary table

Supplementary Table 1. Primer sequences

Primer name	Primer Sequence
Bmal1 forward	TGCAATGTCCAGGAAGTTAGAT
Bmal1 reverse	GTTTGCTTCTGTGTATGGGTT
Per2 forward	ATGCTCGCCATCCACAAGA
Per2 reverse	GCGGAATGGAATGGGAGAAT
Mcub forward	TCACAAGAAAGGTCAAAGCTGC
Mcub reverse	CCAGGAGTACACCCACCAAG
Emre forward	GACGACGATTAACAGGGCAC
Emre reverse	CAGGACTCTGGGCTCTTGTC
Nr1d1 forward	GAGAGGCCATCACAACCTCC
Nr1d1 reverse	ACACCACCTGTGTTGTTATTGG
Fis1 forward	AGGCTCTAAAGTATGTGCGAGG
Fis1 reverse	GGCCTTATCAATCAGGCGTTC
Opa1 forward	CTGCAGGTCCCAAATTGGTT
Opa1 reverse	TCTTTGTCTGACACCTTCCTGT
Mfn1 forward	CCTACTGCTCCTTCTAACCCA
Mfn1 reverse	AGGGACGCCAATCCTGTGA
Rps18 forward	CCCTCTATGGGCTCGAATTT
Rps18 reverse	GGATGTGAAGGATGGGAAGT

II. Supplementary Figures

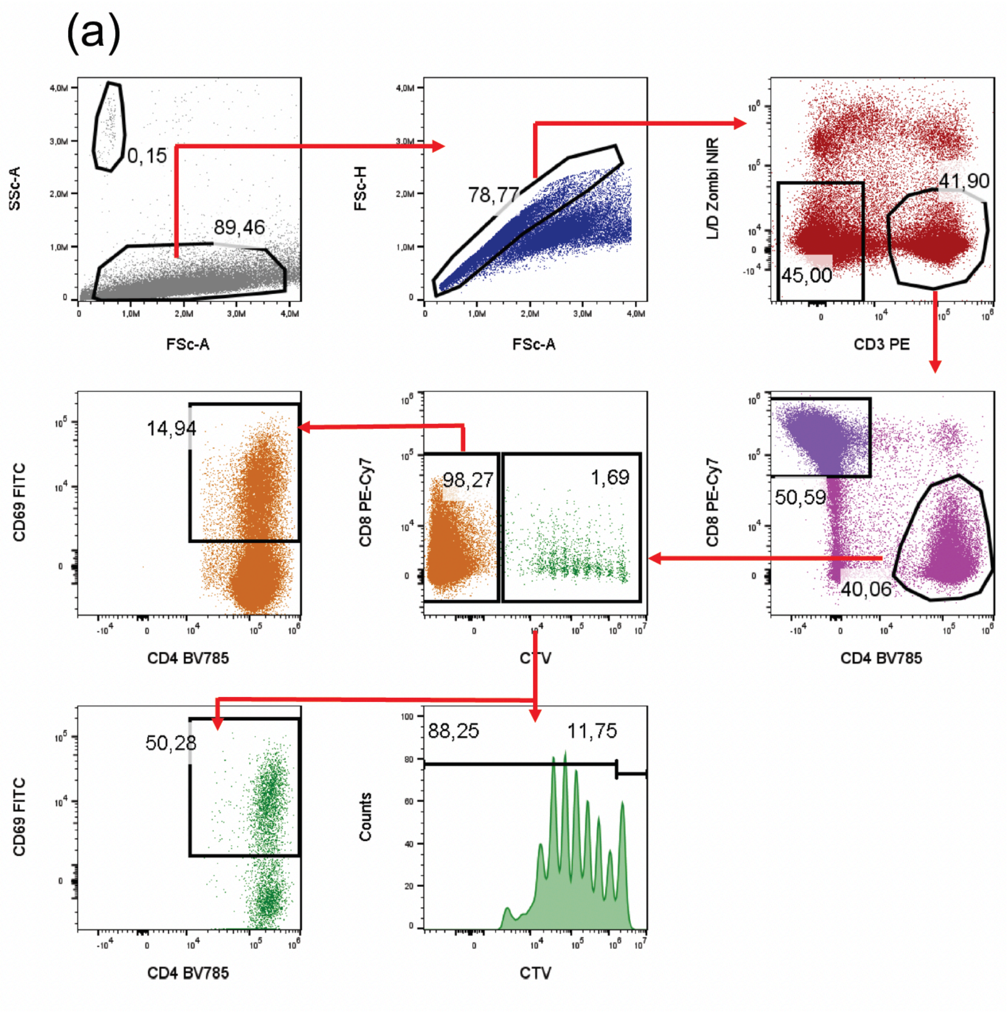

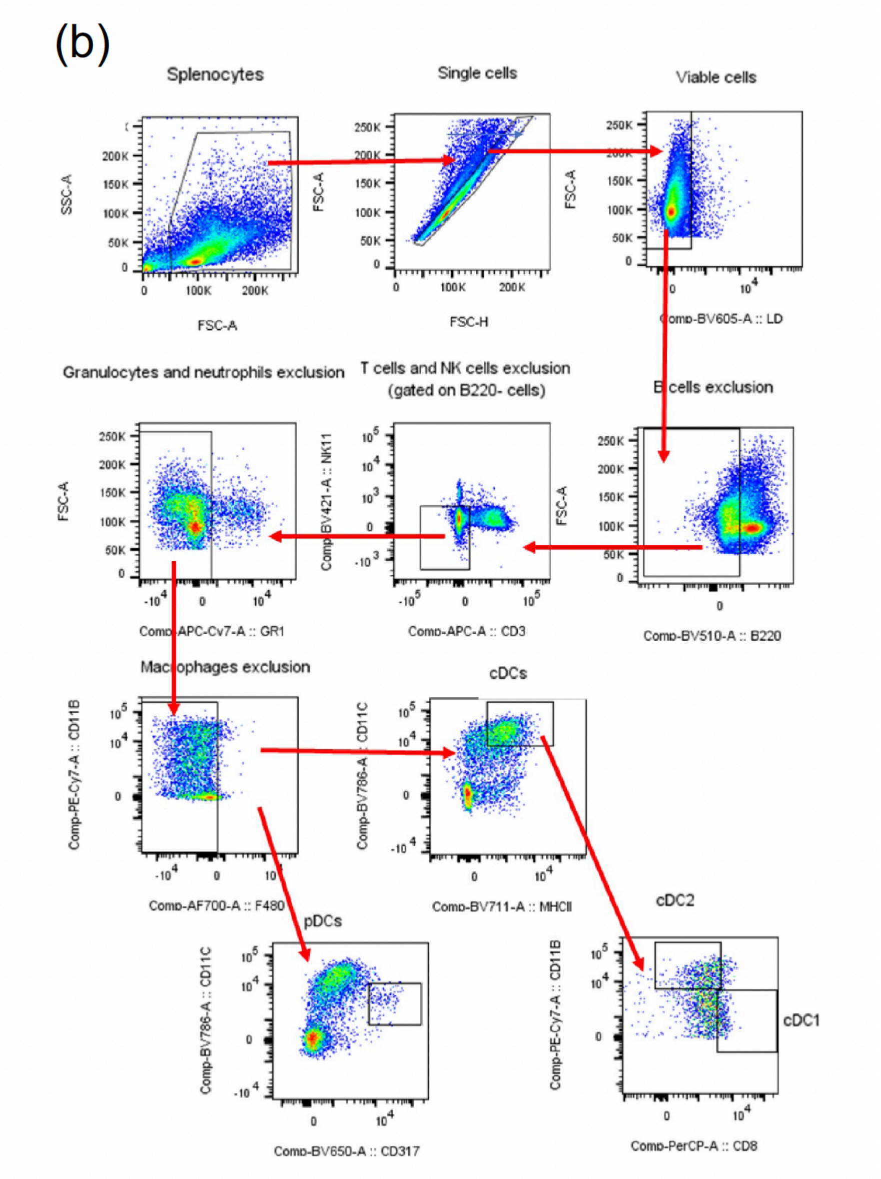

Supplementary Fig. 1. Gating strategies

(**a**) Gating strategy for CTV^{+} stained OT-II CD4 T cells obtained by lymph node harvesting and analysed via flow cytometry (**Fig. 1**) (**b**) Gating strategy for splenic DCs populations obtained by spleen isolation and analysed via flow cytometry (**Fig. 2**).

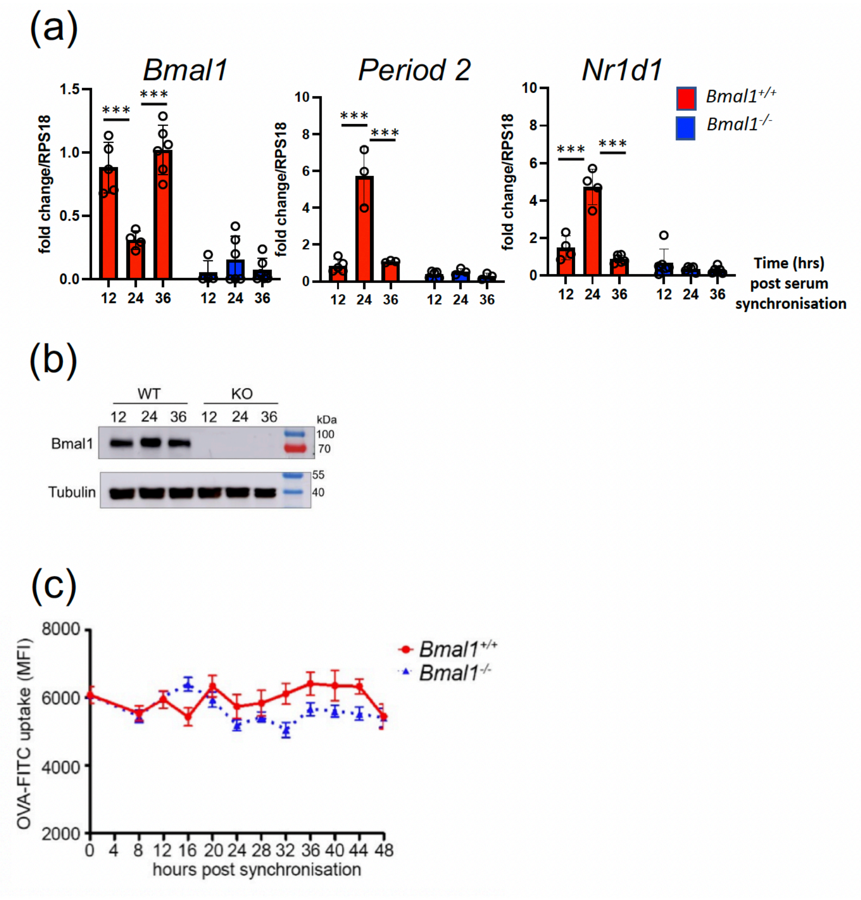

Supplementary Fig. 2. Synchronised DCs produce rhythms in clock gene expression but antigen uptake is not affected by the clock

(**a**) *Bmal1*$^{+/+}$ and *Bmal1*$^{-/-}$ BMDCs were synchronised and mRNA levels of *Bmal1*, *Nr1d1* and *Per2* were analysed by qPCR at designated time points post synchronisation (n=3 biologically independent samples). (**b**) *Bmal1*$^{+/+}$ and *Bmal1*$^{-/-}$ BMDCs were synchronised and subjected to immunoblot analysis for Bmal1 protein at designated time points post synchronisation (n=1 biologically independent sample). (**c**) *Bmal1*$^{+/+}$ and *Bmal1*$^{-/-}$ BMDCs were synchronised and OVA uptake was measured using FITC-OVA at designated time points post synchronisation and quantified by confocal microscopy. (n=20 biologically independent samples)

Data shown is mean with error bars representing ± SEM. Data were compared by one-way ANOVA with Tukey's post-hoc test for multiple comparisons. *** $p<0.001$

Source data are provided as a source data file.

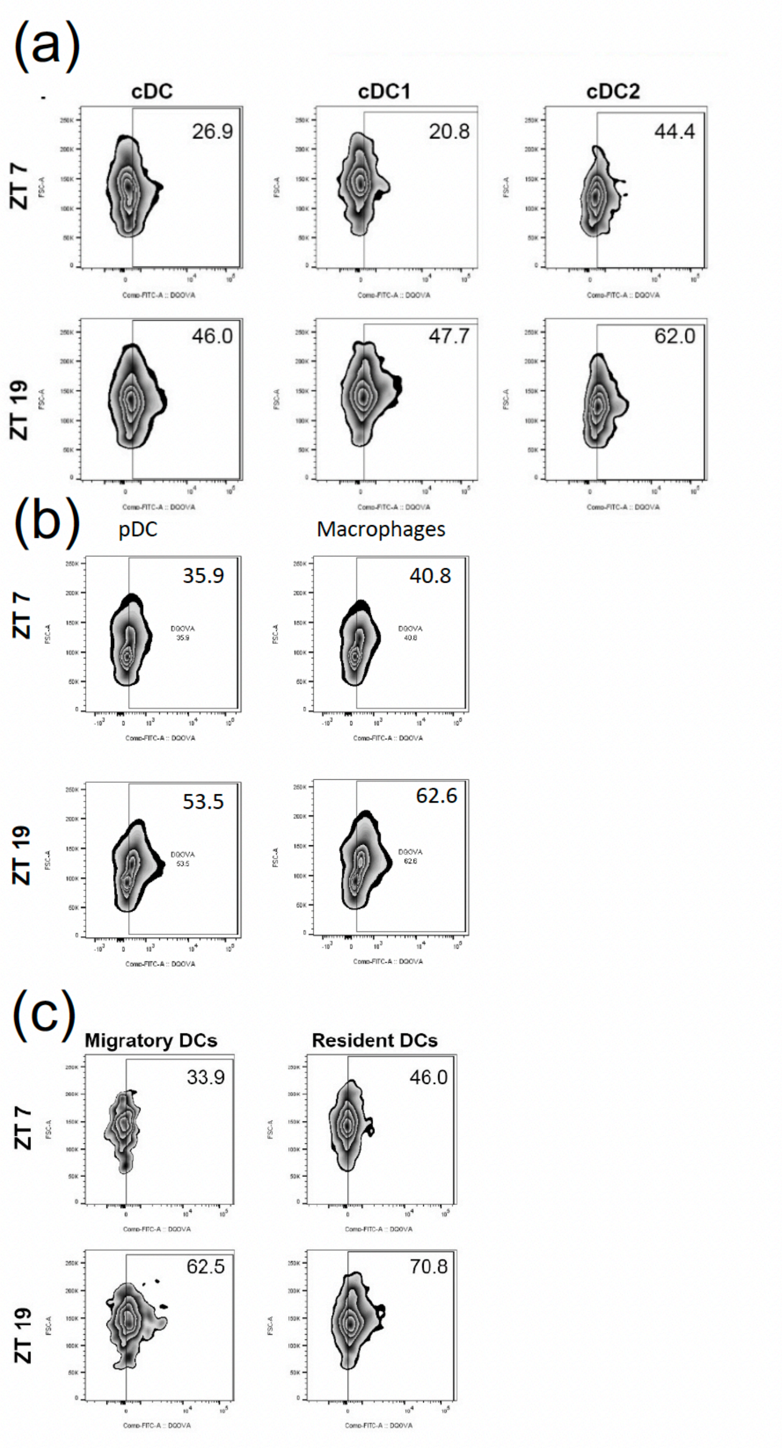

Supplementary Fig. 3. Flow cytometry analysis of DC subsets for antigen processing

(**a-c**) Splenic DCs were expanded by B16-FLT3l cells and spleens were isolated from mice at ZT7 and ZT19. Single cell suspensions were generated, then incubated with DQ-OVA (1 μg/mL) for 60 min prior to staining and characterisation by flow cytometry.

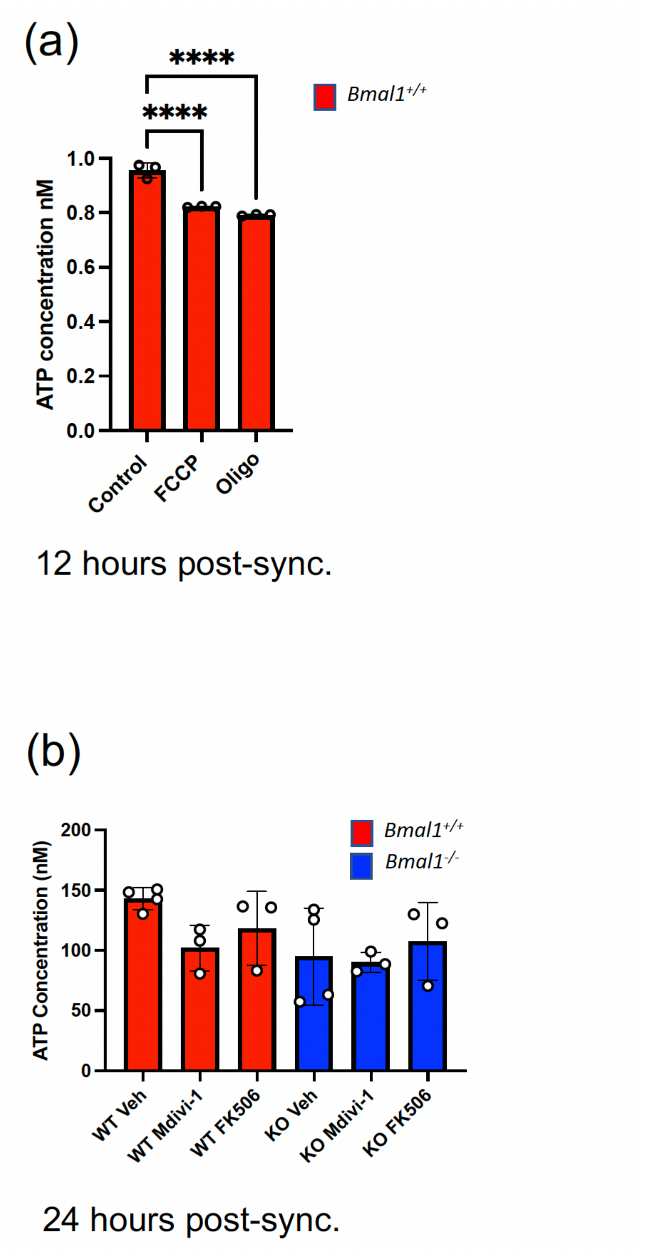

Supplementary Fig. 4. Effect of Oligomycin, FCCP, Mdivi-1 and FK506 on ATP levels

(a) *Bmal1*$^{+/+}$ BMDCs were synchronised and pretreated with FCCP (10 μM), Oligomycin (10 μM) or vehicle for 3 hours and then harvested at 12 hours post synchronisation and ATP measured (n=3 biologically independent samples). **(b)** *Bmal1*$^{+/+}$ and *Bmal1*$^{-/-}$ BMDCs were synchronised and pre-treated with Mdivi-1 (10 μM), FK506 (10 μM) for 12 hours and then harvested at 24 hours post synchronisation and ATP measured. (n=3-4 biologically independent samples).Data shown is mean with error bars representing ± SEM. Data were compared by one-way ANOVA with Tukey's post-hoc test for multiple comparisons. ****$p < 0.0001$. Source data are provided as a source data file.

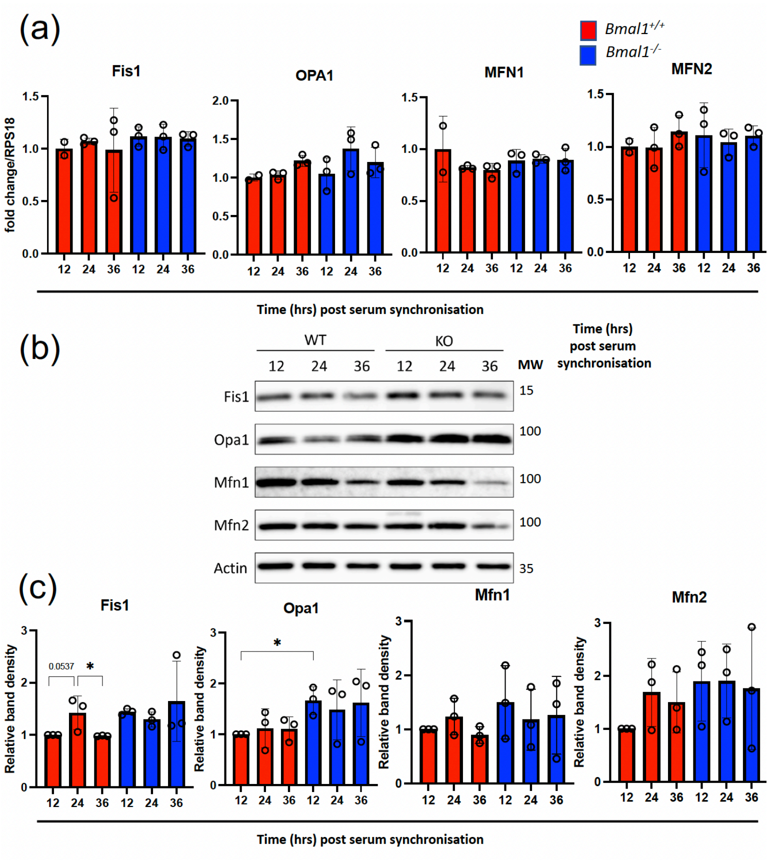

Supplementary Fig. 5. Genes involved in mitochondrial morphology do not display circadian rhythms at mRNA and protein level except for *Fis1*

(**a**) *Bmal1*$^{+/+}$ and *Bmal1*$^{-/-}$ BMDCs were synchronised by serum shock and mRNA levels of genes associated with mitochondrial morphology were analysed by qPCR at 12 hr, 24 hr and 36 hr post synchronisation (n= 2-3 biologically independent samples). *Bmal1*$^{+/+}$ and *Bmal1*$^{-/-}$ BMDCs were synchronised by serum shock and protein levels of genes associated with mitochondrial morphology were analysed by **(b)** immunoblot at 12 hr, 24 hr and 36 hr post synchronisation and (c) bands quantified (n=3 biologically independent samples). Source data are provided as a source data file. Data shown is mean with error bars representing ± SEM. Data were analysed by one-way ANOVA with Tukey's post-hoc test for multiple comparisons. *p<0.05. Source data are provided as a source data file.

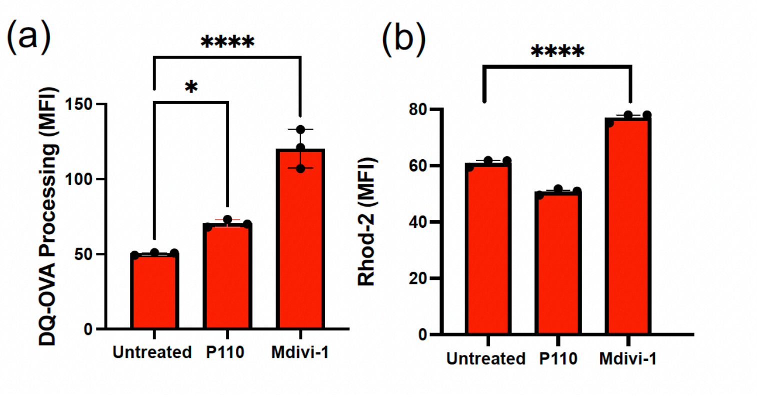

Supplementary Fig. 6. Mitochondrial fission inhibitor P110 does not promote mitochondrial Ca^{++} uptake and is less efficient than Mdivi-1 at promoting antigen processing

Bmal1$^{+/+}$ BMDCs were synchronised by serum shock. (**a**) Antigen processing and (**b**) mitochondrial calcium uptake was quantified at 24 hr post synchronisation in the presence and absence of P110 (1 μM) or Mdivi-1 (10 μM) (n=3 biologically independent samples). Data shown is mean with error bars representing ± SEM. Data were analysed by one-way ANOVA with Tukey's post-hoc test for multiple comparisons. **p<0.05 and **** p<0.0001. Source data are provided as a source data file.

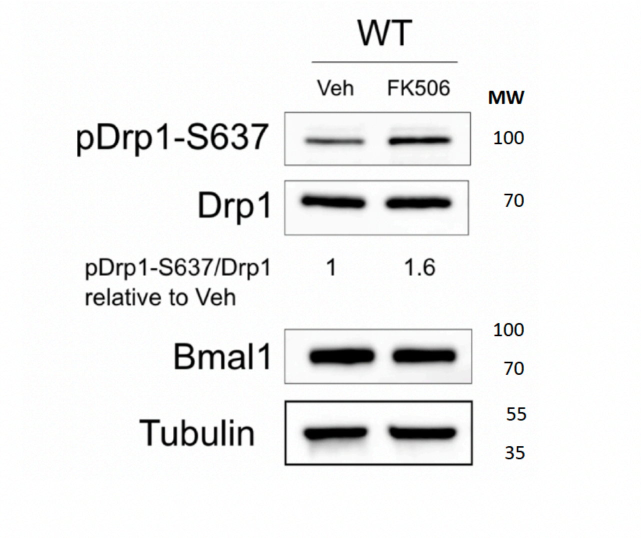

Supplementary Fig. 7. FK506 prevents the dephosphorylation of S637 on DRP1

Bmal1$^{+/+}$ BMDCs were synchronised by serum shock and pre-treated with FK506 (1 μM) at 21 hours post-synchronisation. Cells were lysed for immunoblot analysis at 24 hours post-synchronisation and probed for pDrp1-S637 and BMAL1 (n=1 biologically independent sample).

Uncropped scans of blots and gels

Supplementary Figure 2b

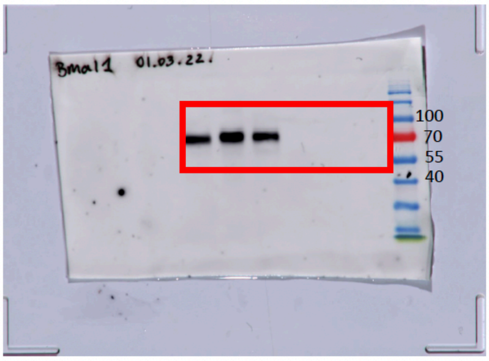

anti-Bmal1 rabbit (Cell Signaling Technology Cat# 14020, RRID:AB_2728705) 1:1000

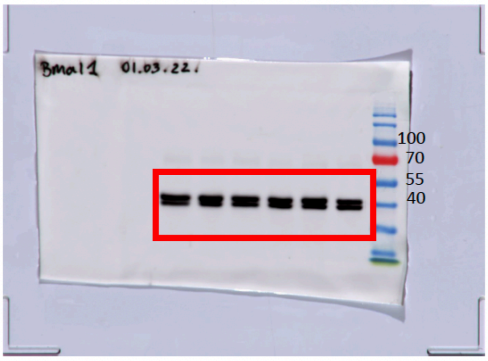

anti-Tubulin mouse (Cell Signaling Technology Cat# 3873, RRID:AB_1904178) 1:1000

Supplementary Figure 5b

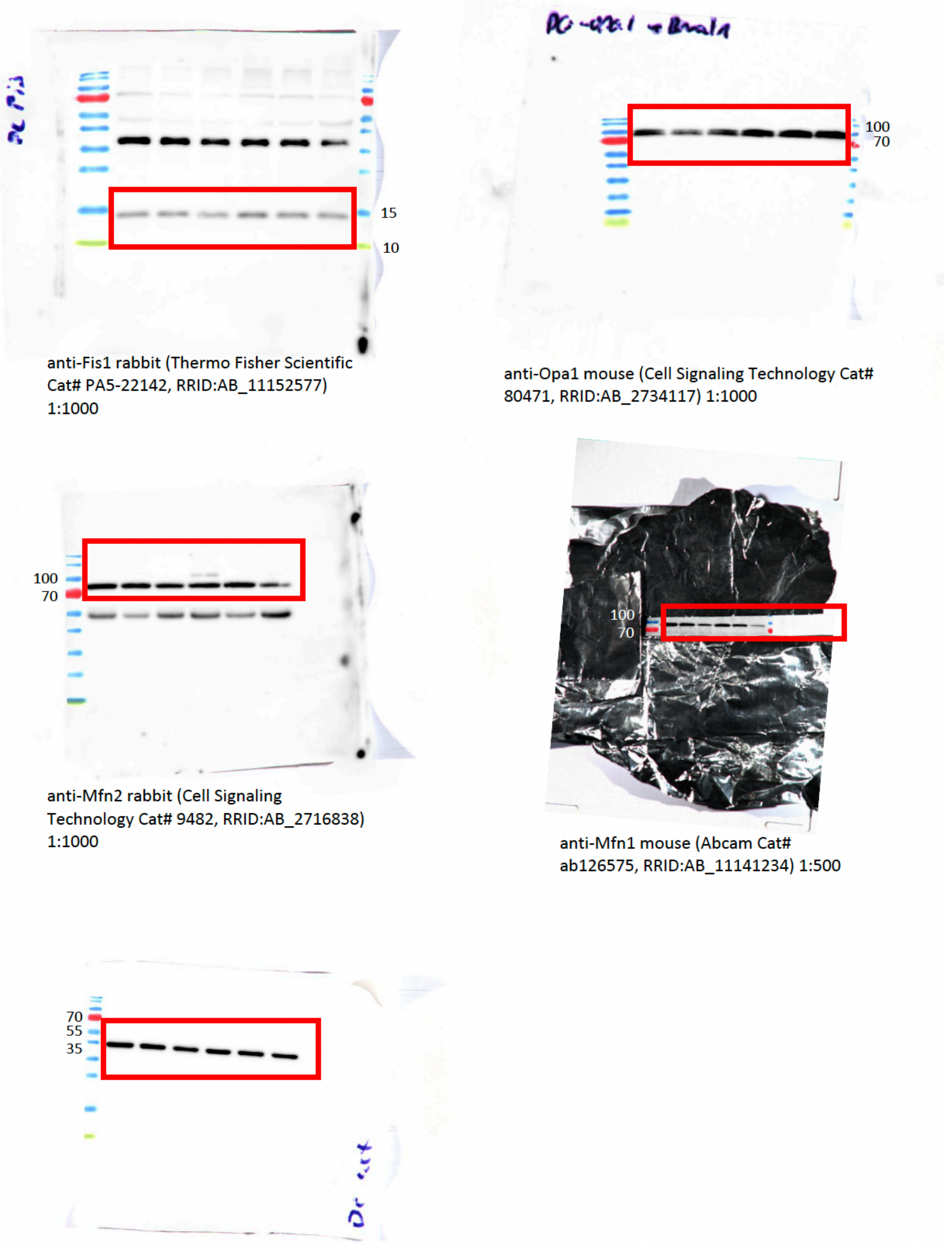

anti-Fis1 rabbit (Thermo Fisher Scientific Cat# PA5-22142, RRID:AB_11152577) 1:1000

anti-Opa1 mouse (Cell Signaling Technology Cat# 80471, RRID:AB_2734117) 1:1000

anti-Mfn2 rabbit (Cell Signaling Technology Cat# 9482, RRID:AB_2716838) 1:1000

anti-Mfn1 mouse (Abcam Cat# ab126575, RRID:AB_11141234) 1:500

anti-β actin mouse (Millipore Cat# MAB1501, RRID:AB_2223041) 1:10000

Supplementary Figure 7

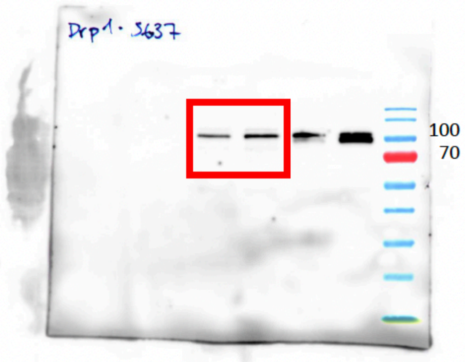

anti-pDrp1(S637) rabbit (Cell Signaling Technology Cat# 4867, RRID:AB_10622027) 1:1000

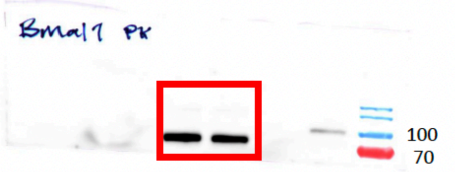

anti-Bmal1 rabbit (Cell Signaling Technology Cat# 14020, RRID:AB_2728705) 1:1000

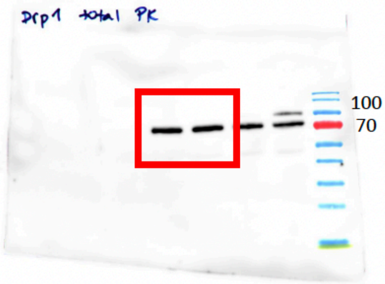

anti-Drp1 rabbit (Cell Signaling Technology Cat# 5391, RRID:AB_11178938) 1:1000

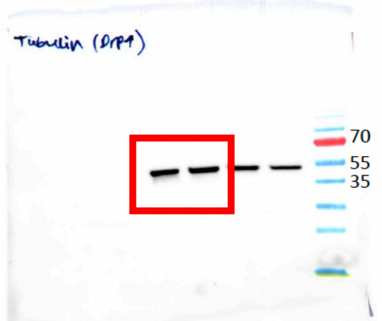

anti-Tubulin mouse (Cell Signaling Technology Cat# 3873, RRID:AB_1904178) 1:1000
