# Supplementary material for: The circadian clock influences T cell responses to vaccination by regulating dendritic cell antigen processing
Source: Nat Commun. 2022 Dec 5;13:7217. doi: 10.1038/s41467-022-34897-z (PMC9722918; doi:10.1038/s41467-022-34897-z)
Supplement: Supplementary file 3 — Description of Additional Supplementary Files [file 41467_2022_34897_MOESM3_ESM.pdf]

Description of Additional Supplementary Files

Supplementary Software 1. Macro and short user guide for mitochondrial size analysis (mitochondrial morphology).
