# Supplementary material for: The circadian clock influences T cell responses to vaccination by regulating dendritic cell antigen processing
Source: Nat Commun. 2022 Dec 5;13:7217. doi: 10.1038/s41467-022-34897-z (PMC9722918; doi:10.1038/s41467-022-34897-z)

A short guide to (semi)automated mitochondria analysis using Fiji (ImageJ)

Ingmar Schoen, RCSI Dublin, ©2021

1. Installation

A.
Download Fiji from https://fiji.sc.
Unpack it.
Start it.

B.
Click "Help > Update…"

C.
We also need to install an additional plugin (only once).
In the "ImageJ Updater" Dialog, click „Manage Update Sites".
Scroll down and check the "IJPB-plugins".
Then click close.

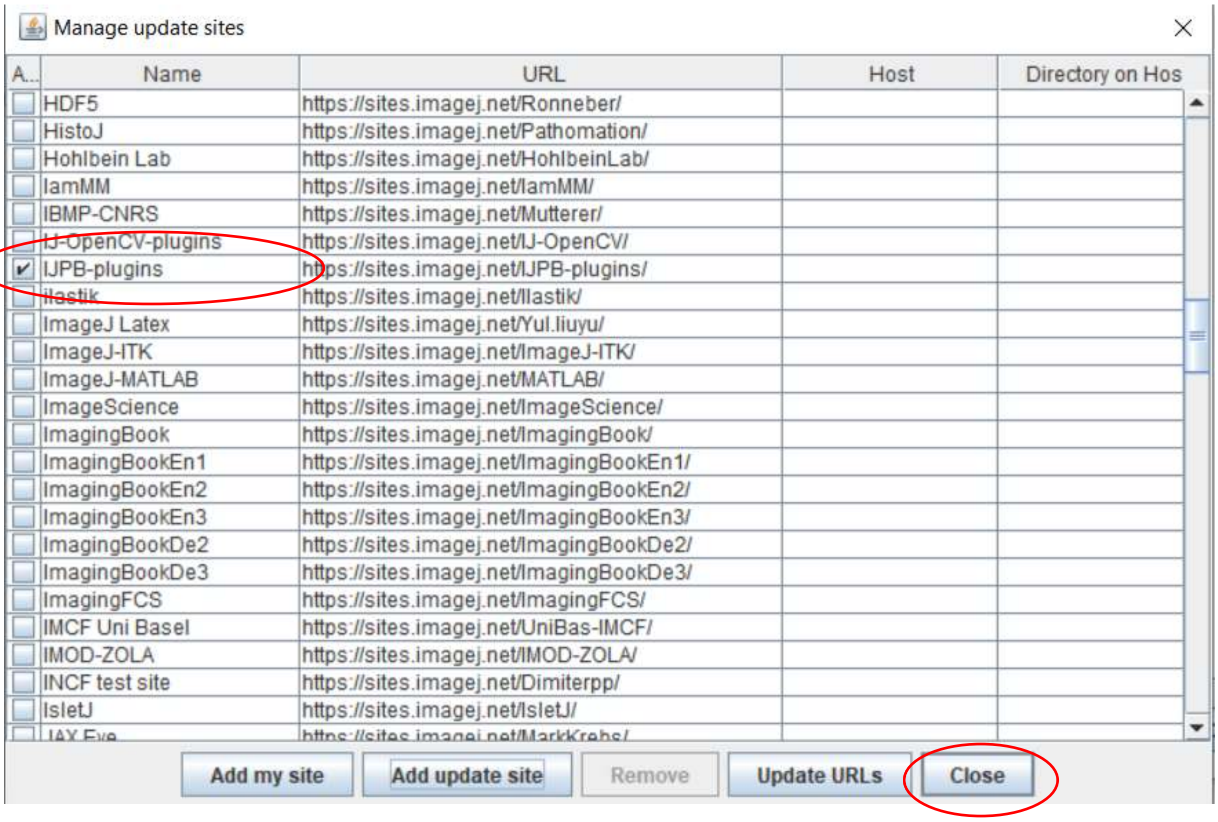

D.
Finish the update by clicking "Apply changes".
Follow the prompts which may require a restart.

Comment:
It is a good idea to always keep your Fiji up to date. Either let Fiji automatically search for updates on startup, or from time to time repeat steps B+D.

2. Set up Fiji for MitoAnalysis

Start Fiji.
Copy the four *.ijm macros (“BatchOpen”, “BatchOutlines”, BatchMito”, BatchAnalyze”) into any folder.
Mark them and drag-drop them onto the Fiji window/info bar:

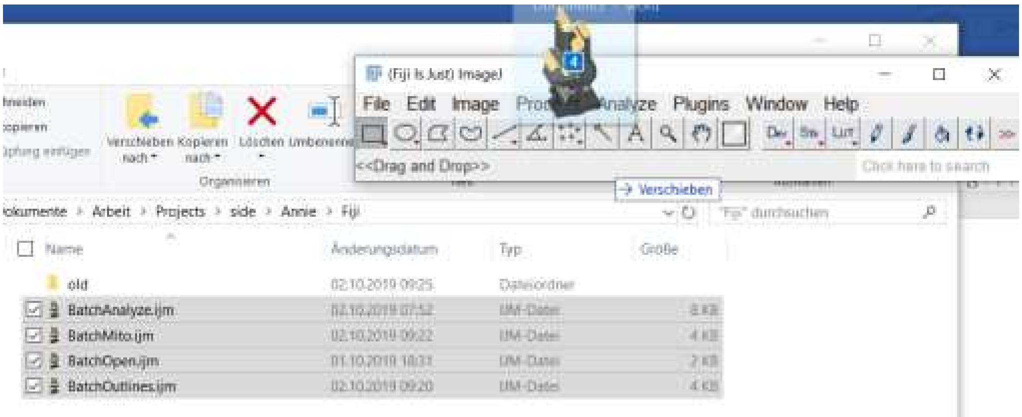

This will open the Script Editor.
Each macro is in a single tab. To switch between them, click on the tab.
To run the macro in the active tab, click “Run”.

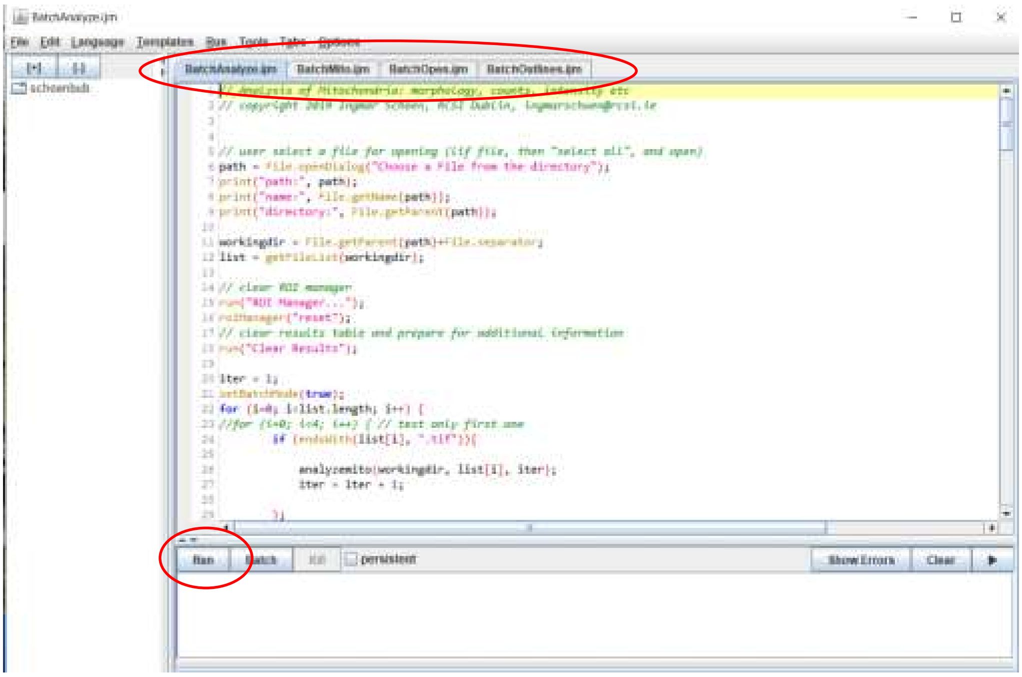

3. Preprocessing of images

Comment:
It is assumed that you have your confocal images in a Bioformats-compatible image format (.lif, *.lsm, *.tif). The container can contain multiple images. It is also assumed that one of the channels contains the mitochondria stainings and that this channel is the same for all images in the container. The “BatchOpen” macro will open one image container and save all mito-images of this container into one folder with the corresponding name.*

Run the “BatchOpen” macro.

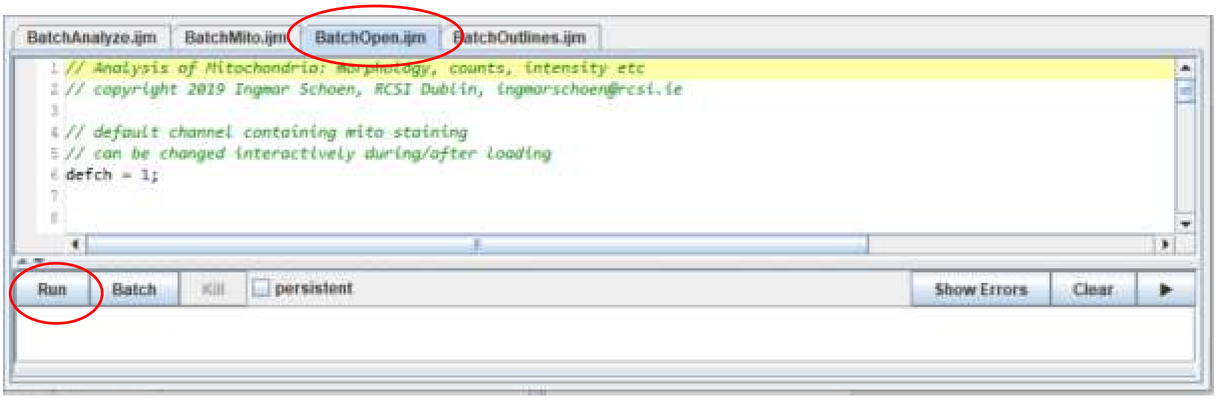

In the “Choose a file” dialog, navigate to the image file and open it.
The macro now loads all images from the container. Then it prompts you to select the channel that contains the mitochondria stain.
Verify which channel this is by navigating through the image. Enter the channel number and click OK.

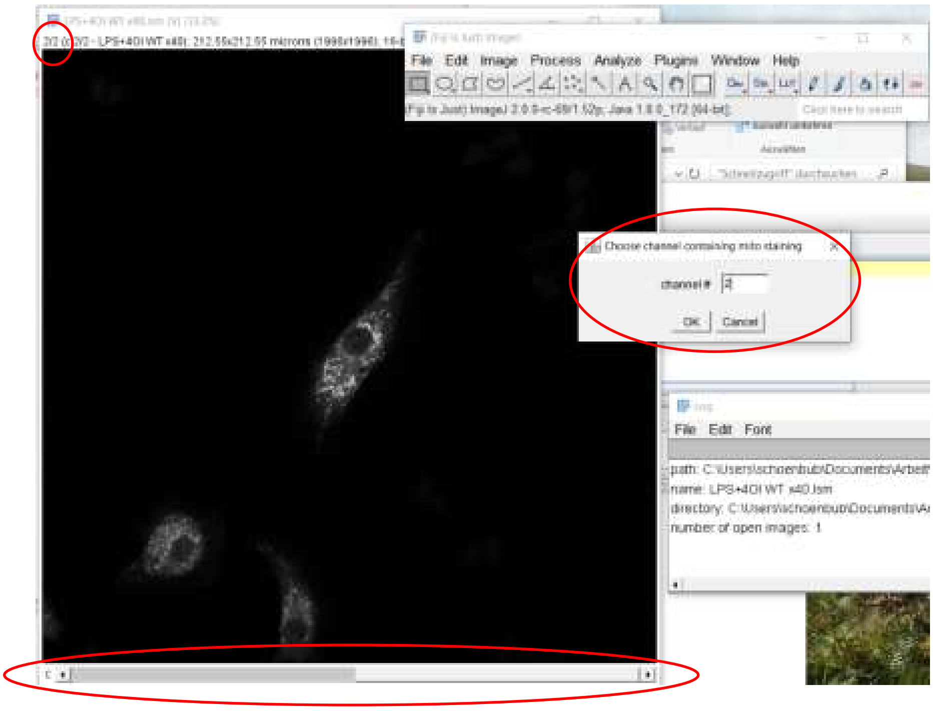

The macro creates a subfolder in the same folder as the image container and saves the mitochondria images as tiff files into it using a naming convention “{name}-mito.tif”.

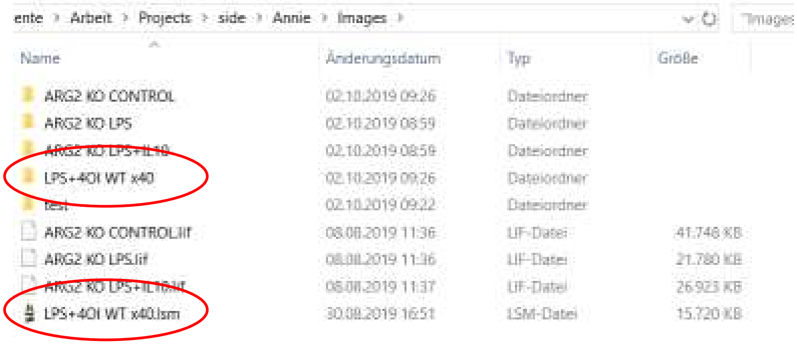

4. Segmentation of cells

Run the “BatchOutlines” macro.

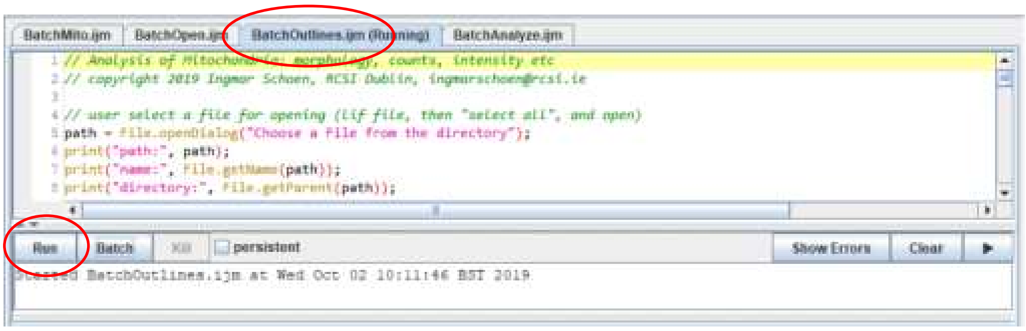

In the “Choose a file” dialog, navigate to the first tiff file in the image folder and open it.

A dialog window asks for the method of segmentation. Default is “NoOutlines” (takes whole image). Alternatively, select “FromImage” to estimate outlines from the WPB stainings (only works okay for isolated cells). Press OK.

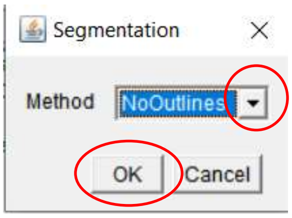

The macro then automatically processes the image and saves the cell mask as *.png file using the naming convention “{name of tiff file}_outline.png”. It does this for all tiff tiles in the folder. The progress can be seen in the “Log” window.

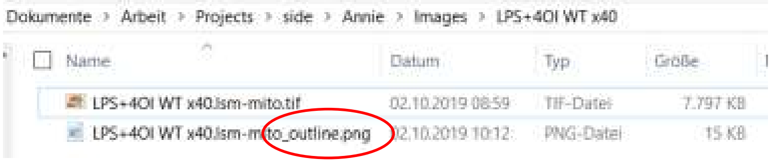

Comment:

This cell segmentation is not perfect at all. It is strongly recommended to check the quality of the segmented cells and to post-process them manually to remove background objects, divide touching cells, reconnect false divisions, and to eliminate cells at the edge which should be excluded from the analysis. This can be easily done at the image processing computer of the SchoenLab using the “EditCells” GUI (MATLAB). For guidance, please ask Ingmar or Martin.

5. Segmentation of mitochondria

Comment:

The segmentation of mitochondria is a compromise between separating touching ones but not splitting elongated ones. This can be controlled by a “prominence” parameter. Make sure that you only compare results that have been processed using the same parameter value.

Enter a “prom” parameter (line 7). Larger values mean less splitting. Save the macro with the parameter that you use by default.

Run the “BatchMito” macro.

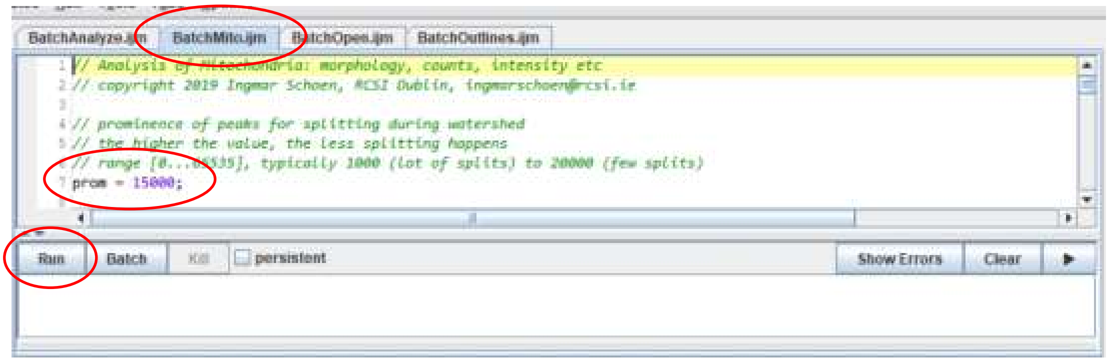

In the “Choose a file” dialog, navigate to the first tiff file in the image folder and open it.
The macro then automatically processes the image and saves the mitochondrial mask as *.png file using the naming convention “{name of tiff file}_objs.png”.
It does this for all tiff tiles in the folder. The progress can be seen in the “Log” window. When it is finished, it might display the last mask (or not).

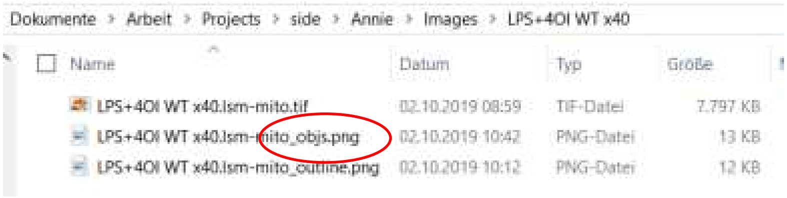

5. Statistical analysis of mitochondria

Comment:
Make sure that the cell segmentation has been revised before running the analysis.
Make sure that all files (.tif, *_objs.png, *_outline.png) are present in the folder.*

Run the “BatchAnalyze” macro.

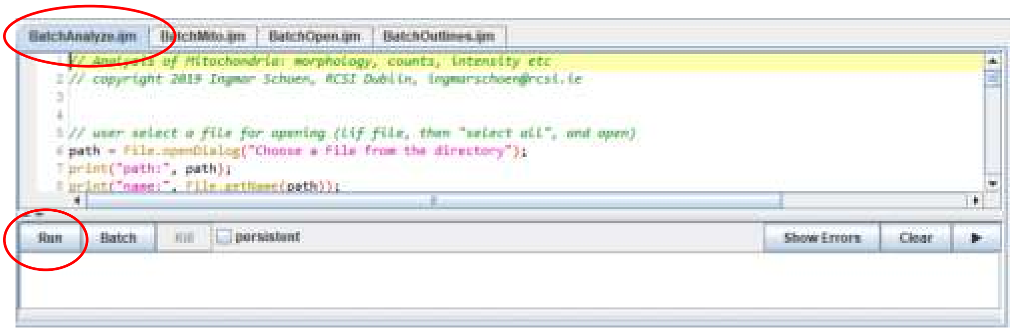

In the “Choose a file” dialog, navigate to the first tiff file in the image folder and open it.
The macro then automatically processes the images. You might observe a hectic activity of the “Results”, “ResultsMito”, and “ResultsCells” windows during processing. The “Log” window also shows the progress.

The macro saves two result tables as comma separated value *.csv files in the parent folder, aside the original image container file using the naming convention “{original name}_CellStatistics.csv” and “{original name}_MitoStatistics.csv”.

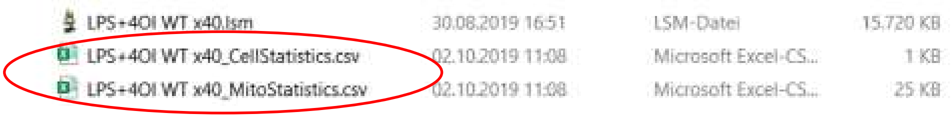

It also replaces the *.tif files in the subfolder with a version of themselves that contains the outlines of cells and mitochondria as an overlay.

6. Analysis of results

A. Cell statistics

Open the "{original name}_CellStatistics.csv" file using your favourite program (Excel, Prism, etc.).

The columns contain different parameters. The rows contain different cells.

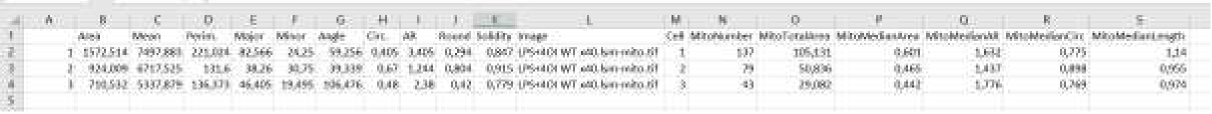

	A	B	C	D	E	F	G	H	I	J	K	L	M	N	O	P	Q	R	S
1		Area	Mean	Perim.	Major	Minor	Angle	Circ.	AR	Round	Solidity	Image	Cell	MitoNumber	MitoTotalArea	MitoMedianArea	MitoMedianAR	MitoMedianCirc	MitoMedianLength
2	1	1572,514	7497,881	221,024	42,566	24,25	59,256	0,405	3,405	0,294	0,847	LPS+4OI WT x40.lsm-mito.tif	1	137	105,131	0,601	1,632	0,775	1,14
3	2	924,009	6717,525	131,6	38,26	30,75	39,339	0,67	1,244	0,804	0,915	LPS+4OI WT x40.lsm-mito.tif	2	79	50,836	0,465	1,437	0,898	0,955
4	3	710,532	5337,879	136,373	46,405	19,495	106,476	0,48	2,38	0,42	0,779	LPS+4OI WT x40.lsm-mito.tif	3	43	29,082	0,442	1,776	0,769	0,974
5																			

The meaning of the entries is as follows:

Column	*Identifier*	*Unit*	*Description*
A		1	Running number
B	Area	μm^2	Cell area
C	Mean	a.u.	Mean intensity of the mitostaining inside the cell outline
D	Perim.	µm	Cell perimeter (length of cell outline)
E	Major	µm	Cell length (major axis of fitting ellipse)
F	Minor	µm	Cell width (minor axis of fitting ellipse)
G	Angle	° deg	Cell orientation (relative to X axis)
H	Circ.	1	Cell circularity (the ratio between a perimeter of a perfect circle of the same area and the cell perimeter).<br>*Circularity=1 is a circle, Circularity=0 is a cell with a lot of outline but little area.*<br>*This measure contains both elongation and irregularity/tortuosity.*
I	AR	1	Cell aspect ratio (cell length/cell width).<br>*AR=1 is a circle, AR>1 is elongated.*<br>*AR is a good measure of cell elongation.*
J	Round	1	I don't know (look up ImageJ docu)
K	Solidity	1	I don't know (look up ImageJ docu)
L	Image	Text	Name of the original image
M	Cell	1	Cell ID in the original image
N	MitoNumber	1	Total number of mitochondria in this cell
O	MitoTotalArea	μm^2	Total area of mitochondria in this cell.<br>*Could represent mitochondrial volume.*<br>*Might correlate with cell area.*
P	MitoMedianArea	μm^2	Median area of mitochondria in this cell.
Q	MitoMedianAR	1	Median aspect ratio of mitochondria in this cell.
R	MitoMedianCirc	1	Median circularity of mitochondria in this cell.
S	MitoMedianLength	µm	Median length of mitochondria in this cell.
T	MitoMedianIntensity	1	Median mean intensity of mitochondria in this cell.

Entries B…K concern the cell, entries N…T the mitochondria.
Columns L,M can be used to find each and every cell in the original image (see **C** below).

C. Mitochondria statistics

Import the “{original name}_MitoStatistics.csv” file using your favourite program (Excel, Prism, etc.).

The columns contain different parameters. The rows contain individual mitochondria.

	A	B	C	D	E	F	G	H	I	J	K	L	M	N	O	P	Q	R	S	T
1		Area	Mean	Perim.	Major	Minor	Angle	Circ.	Feret	FeretX	FeretY	FeretAngle	MinFeret	AR	Round	Solidity	Image	Cell	WPBinCell	WPBinImage
2	1	1.843	10901.54	5.696	3.176	1.077	52.824	0.713	2.347	27	14	51.843	1.353	2.021	0.495	0.841	1mM_ctrl_	1	1	1
3	2	0.365	8442.048	2.173	0.834	0.557	95.113	0.971	0.884	51	0	116.565	0.527	1.497	0.668	0.933	1mM_ctrl_	1	2	2
4	3	1.772	11008.08	7.868	3.324	0.679	95.514	0.36	3.261	118	0	104.036	0.845	4.9	0.204	0.736	1mM_ctrl_	1	3	3
5	4	3.248	9594.316	12.477	3.22	1.284	107.304	0.262	4.071	228	0	119.055	1.641	2.507	0.399	0.636	1mM_ctrl_	1	4	4
6	5	0.556	6437.281	2.809	1.057	0.669	54.946	0.885	1.215	261	7	49.399	0.766	1.58	0.633	0.901	1mM_ctrl_	1	5	5
7	6	1.442	24342.52	4.641	1.594	1.152	90.596	0.841	1.793	329	0	107.103	1.186	1.384	0.723	0.883	1mM_ctrl_	1	6	6
8	7	0.33	5606.316	2.205	0.945	0.445	159.68	0.853	1.004	449	1	156.801	0.527	2.126	0.47	0.844	1mM_ctrl_	1	7	7
9	8	0.886	7645.608	4.346	1.761	0.641	88.005	0.589	1.793	465	13	72.897	0.659	2.749	0.364	0.843	1mM_ctrl_	1	8	8
10	9	0.504	15158.9	3.259	1.416	0.453	3.323	0.596	1.474	522	2	10.305	0.527	3.125	0.32	0.853	1mM_ctrl_	1	9	9
11	10	0.851	12423.59	3.446	1.37	0.791	29.431	0.901	1.503	577	7	37.875	0.884	1.733	0.577	0.925	1mM_ctrl_	1	10	10
12	11	1.181	9688.059	5.464	1.688	0.891	99.584	0.497	1.887	623	13	65.225	1.054	1.894	0.528	0.735	1mM_ctrl_	1	11	11
13	12	1.424	9731.488	6.364	1.812	1.001	22.995	0.442	2.347	674	11	38.157	1.45	1.811	0.552	0.686	1mM_ctrl_	1	12	12
14	13	1.702	7995.918	7.9	2.248	0.965	86.641	0.343	2.717	77	2	112.834	1.689	2.328	0.43	0.541	1mM_ctrl_	1	13	13
15	14	1.598	11608.12	4.937	1.561	1.303	96.348	0.824	1.667	200	13	71.565	1.435	1.198	0.835	0.868	1mM_ctrl_	1	14	14
16	15	0.747	6446.14	4.673	1.597	0.595	80.774	0.43	1.758	905	1	102.995	0.866	2.684	0.373	0.593	1mM_ctrl_	1	15	15
17	16	0.347	10934.35	2.205	0.927	0.477	19.141	0.898	1.004	140	6	23.199	0.527	1.942	0.515	0.87	1mM_ctrl_	1	16	16
18	17	2.414	15406.6	9.423	2.632	1.168	72.311	0.342	3.36	157	27	78.69	1.787	2.253	0.444	0.607	1mM_ctrl_	1	17	17
19	18	0.469	11172.7	2.655	1.09	0.548	17.67	0.836	1.179	129	8	26.565	0.625	1.988	0.503	0.831	1mM_ctrl_	1	18	18
20	19	1.094	5785.683	4.905	1.463	0.952	105.016	0.572	1.718	275	5	122.471	1.186	1.536	0.651	0.737	1mM_ctrl_	1	19	19
21	20	1.042	8021.867	3.741	1.481	0.896	9.926	0.936	1.543	888	9	19.983	0.923	1.653	0.605	0.909	1mM_ctrl_	1	20	20
22	21	0.886	12080.47	3.896	1.556	0.725	159.037	0.734	1.651	537	4	151.39	0.884	2.147	0.466	0.872	1mM_ctrl_	1	21	21
23	22	0.886	11027	3.523	1.397	0.807	101.344	0.897	1.474	593	4	116.565	0.791	1.731	0.578	0.944	1mM_ctrl_	1	22	22

The meaning of the entries is as follows:

Column	*Identifier*	*Unit*	*Description*
A		1	Running number
B	Mean	1	Mean Mitochondrion staining intensity
C	Area	μm^2	Mitochondrion area
D	Perim.	µm	Mitochondrion perimeter (length of 7itochondrion outline)
E	Major	µm	Mitochondrion length (major axis of fitting ellipse).
F	Minor	µm	Mitochondrion width (minor axis of fitting ellipse)
G	Angle	° deg	Mitochondrion orientation (relative to X axis)
H	Circ.	1	Mitochondrion circularity (the ratio between a perimeter of a perfect circle of the same area and the cell perimeter). *Circularity=1 is a circle, Circularity=0 is a very tortuous and/or elongated mitochondrion.*
I	Feret	µm	Mitochondrion length (by Feret diameter = longest line connecting two points on the outline and contained within it).
J	FeretX	µm	Mitochondrion Feret diameter in X.
K	FeretY	µm	Mitochondrion Feret diameter in X.
L	FeretAngle	° deg	Mitochondrion Feret diameter orientation.
M	MinFeret	µm	Minimum mitochondrion Feret diameter.
N	AR	1	Mitochondrion aspect ratio (length/width). *AR=1 is a circle, AR>1 is elongated. AR is a good measure of elongation.*
O	Round	1	Mitochondrion roundness (I don’t know what this is, look up ImageJ docu)
P	Solidity	1	Mitochondrion solidity (I don’t know what this is, look up ImageJ docu)
Q	Image	Text	Name of the original image

R	Cell	1	Cell ID in the original image that contained this mitochondrion.
S	MitoinCell	1	ID of mitochondrion in this cell
T	MitoinImage	1	Running number of mitochondria in image

Commment:
For **<u>mitochondria length</u>**, the Feret diameter (column I) usually is a more accurate measure than the major axis of the fitting ellipse (column E) which tends to underestimate the length systematically, especially for thin and slightly curved morphologies.

Columns Q,R,S can be used to find each and every mitochondrion in the original image (see **C** below).

Columns J-M have no deeper meaning.

<u>**C.** Inspection of outlines.</u>

Open one of the processed *.tif files (subfolder) in Fiji. You should see yellow outlines and a lot of labels on the image.

If the “ROI Manager” is open or not empty, clear it by clicking “Deselect” and then “Delete”.

Click “Image>Overlay>To ROI manager”. *This should populate the ROI manager with cell outlines (“cell#”, top rows) and mitochondria outlines (“cell#-mito##”, below, scroll down). The nomenclature is the same as in the result *.csv files (se above).*

Uncheck “Labels” for a better overview.

You can then click on any ROI in the manager to highlight it in the outline in the image. The active ROI is shown in cyan if the “Show All” checkbox is checked and yellow otherwise.

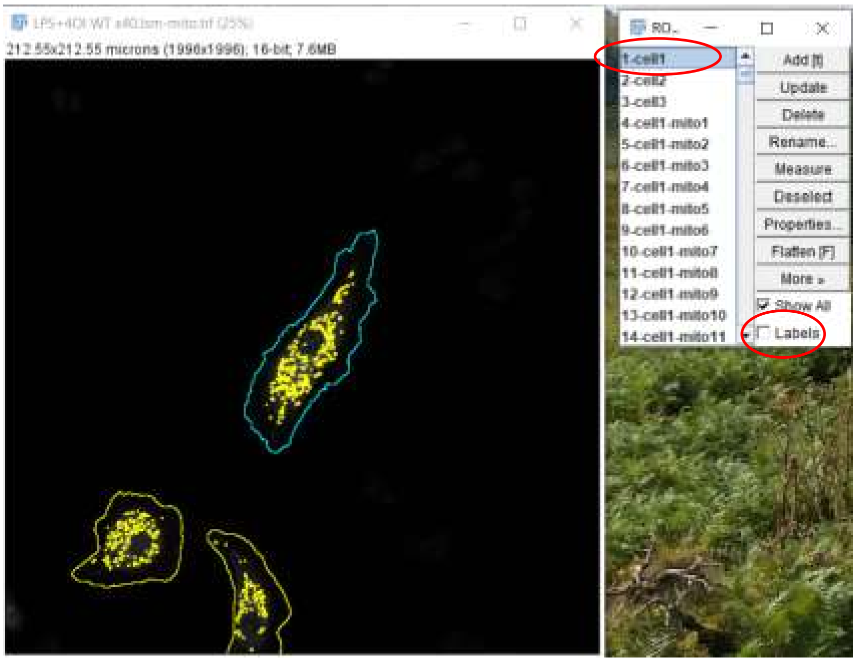

If you want to **export an image** with the overlay for e.g. a publication or presentation,
- adjust the appearance of the raw image as desired, incl contrast and LUT.
- the colour of the outlines can be changed in the “Edit>Options>Colors…” settings dialog.
- convert to RGB by “Image>Type>RGB Color”.
- to burn the overlay into the image, use “Image>Overlay>Flatten”. *This opens up a new window. From here on, the appearance of the outlines cannot be changed independently from the image anymore.*
- evtl. crop the image as desired
- evtl. insert scale bar by “Analyse>Tools>Scale Bar…”.
- save your image. *Do NOT save the RGB tif file under the original name in the subfolder so that the original file is not replaced.*

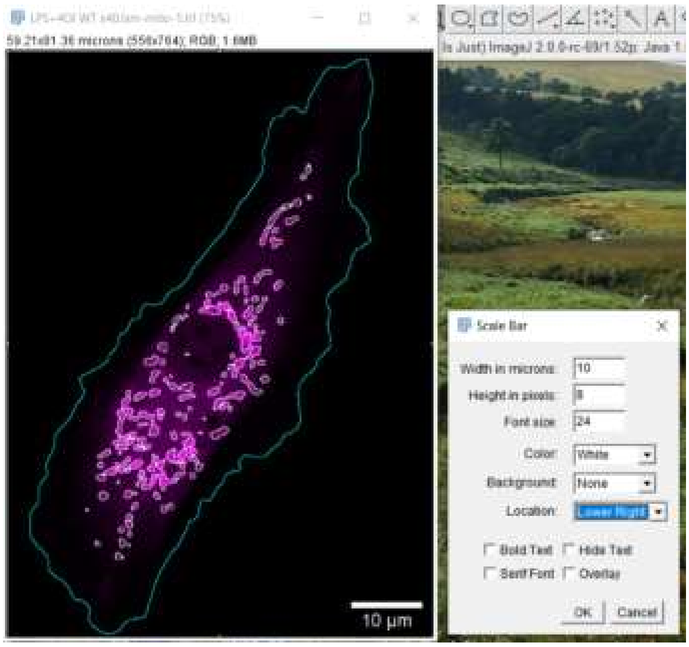

Supplement: Supplementary file 4 — Supplementary Software 1 [file 41467_2022_34897_MOESM4_ESM.zip › Supplementary Software 1/Short_Guide semi automated mito.pdf]
