# Supplementary material for: The circadian clock influences T cell responses to vaccination by regulating dendritic cell antigen processing
Source: Nat Commun. 2022 Dec 5;13:7217. doi: 10.1038/s41467-022-34897-z (PMC9722918; doi:10.1038/s41467-022-34897-z)
Supplement: Supplementary file 5 — Reporting Summary [file 41467_2022_34897_MOESM5_ESM.pdf]

nature portfolio

Corresponding author(s): Annie Curtis

Last updated by author(s): 24th October 2022

Reporting Summary

Nature Portfolio wishes to improve the reproducibility of the work that we publish. This form provides structure for consistency and transparency in reporting. For further information on Nature Portfolio policies, see our Editorial Policies and the Editorial Policy Checklist.

Statistics

For all statistical analyses, confirm that the following items are present in the figure legend, table legend, main text, or Methods section.

n/a	Confirmed	
☐	☒	The exact sample size (*n*) for each experimental group/condition, given as a discrete number and unit of measurement
☐	☒	A statement on whether measurements were taken from distinct samples or whether the same sample was measured repeatedly
☐	☒	The statistical test(s) used AND whether they are one- or two-sided<br>*Only common tests should be described solely by name; describe more complex techniques in the Methods section.*
☐	☒	A description of all covariates tested
☐	☒	A description of any assumptions or corrections, such as tests of normality and adjustment for multiple comparisons
☐	☒	A full description of the statistical parameters including central tendency (e.g. means) or other basic estimates (e.g. regression coefficient) AND variation (e.g. standard deviation) or associated estimates of uncertainty (e.g. confidence intervals)
☐	☒	For null hypothesis testing, the test statistic (e.g. *F*, *t*, *r*) with confidence intervals, effect sizes, degrees of freedom and *P* value noted<br>*Give P values as exact values whenever suitable.*
☒	☐	For Bayesian analysis, information on the choice of priors and Markov chain Monte Carlo settings
☒	☐	For hierarchical and complex designs, identification of the appropriate level for tests and full reporting of outcomes
☒	☐	Estimates of effect sizes (e.g. Cohen's *d*, Pearson's *r*), indicating how they were calculated

Our web collection on statistics for biologists contains articles on many of the points above.

Software and code

Policy information about availability of computer code

Data collection	Applied Biosystems 7500 or 7900 HT was used to collect RT-PCR data<br>XFe96 analyzer (Agilent) was used to perform metabolic assays.<br>Amersham Imager 600 was used to collect western blot images.<br>Leica SP8 scanning confocal microscopy was used to get confocal images.<br>BD Biosciences FACSCanto II cytometer or Cytek Biosciences Aurora spectral analyser was used for flow cytometry data<br>VICTOR3 Multilabel Plate Reader was used for ELISAs.
Data analysis	GraphPad PRISM 8 or 9 was used to perform statistical analysis and plot results.<br>ImageStudioLite was used to perform western blot quantifications.<br>7500 SDS v1.4.1 was used to analyze RT-PCR data.<br>FlowJo v10 software was used for FACs measurements.<br>ImageJ v1.53 was used to quantify confocal data.<br>Stata 14 statistical software for cosinor curve circadian analysis<br>MetaCycle v3.6.1 used for analysis of circadian periodicity

For manuscripts utilizing custom algorithms or software that are central to the research but not yet described in published literature, software must be made available to editors and reviewers. We strongly encourage code deposition in a community repository (e.g. GitHub). See the Nature Portfolio guidelines for submitting code & software for further information.

Data

Policy information about availability of data

All manuscripts must include a data availability statement. This statement should provide the following information, where applicable:

- Accession codes, unique identifiers, or web links for publicly available datasets
- A description of any restrictions on data availability
- For clinical datasets or third party data, please ensure that the statement adheres to our policy

The authors declare that all data are available in the article and supplementary information files.

Human research participants

Policy information about studies involving human research participants and Sex and Gender in Research.

Reporting on sex and gender	N/A
Population characteristics	N/A
Recruitment	N/A
Ethics oversight	N/A

Note that full information on the approval of the study protocol must also be provided in the manuscript.

Field-specific reporting

Please select the one below that is the best fit for your research. If you are not sure, read the appropriate sections before making your selection.

☒ Life sciences ☐ Behavioural & social sciences ☐ Ecological, evolutionary & environmental sciences

For a reference copy of the document with all sections, see nature.com/documents/nr-reporting-summary-flat.pdf

Life sciences study design

All studies must disclose on these points even when the disclosure is negative.

Sample size	For the in vivo studies our preliminary data suggested that we would be able to see a significant difference (90% power to detect a significant difference) with n=3. We calculated if the effect was substantially smaller (1/2 the size, cohen's d=3.5) this would require n=4, and if the effect size was to be substantially smaller (1/2 the size, cohen's d=1.75) this would require n=10. Based on this, we used n=6 in our in vivo studies which was also based on previous studies in the literature (https://doi.org/10.1073/pnas.1905080116) . No previous power calculations were performed for in vitro experiments. We used standard in vitro sample size of n=3 biological for BMDC derived from mice.
Data exclusions	Data was not excluded.
Replication	Biological replicates of at least 3 were used for BMDC experiments. For metabolic assay studies each biological replicate had at least 3 technical replicates Each figure contains details of experimental replicates in the figure legend that were reproduced successfully.
Randomization	For in vitro studies, both male and female mice of C57Bl/6J background, chosen at random, were used at 6-10 weeks of age for WT BMDC generation and comparisons were only made between the same sex. Bmal1+/+ BMDC generated from control Lyz2Cre (Bmal1myeloid+/+) mice were compared to Bmal1-/- BMDC from Bmal1LoxP/LoxP::Lyz2Cre (Bmal1myeloid−/−) mice. Mice (both male and female) were 6-10 weeks old and comparisons were also made between the same gender.<br><br>For the in vivo study, males OT-II mice, chosen at random, were used at 6-10 weeks of age for T cell generation and adoptively transferred into male C57Bl/6J WT mice (6-10 weeks of age) which were randomly assigned into either the ZT7 or ZT19 groups .
Blinding	Blinding was not performed in the in vivo study. The reason for this is that ZT7 mice were immunised in the light and ZT19 mice were immunised in the dark , as such blinding to the different groups was not possible.

Reporting for specific materials, systems and methods

We require information from authors about some types of materials, experimental systems and methods used in many studies. Here, indicate whether each material, system or method listed is relevant to your study. If you are not sure if a list item applies to your research, read the appropriate section before selecting a response.

Materials & experimental systems

n/a	Involved in the study	
☐	☒	Antibodies
☐	☒	Eukaryotic cell lines
☒	☐	Palaeontology and archaeology
☐	☒	Animals and other organisms
☒	☐	Clinical data
☒	☐	Dual use research of concern

Methods

n/a	Involved in the study	
☒	☐	ChIP-seq
☐	☒	Flow cytometry
☒	☐	MRI-based neuroimaging

Antibodies

Antibodies used	Flow Cytometry Antibodies<br>CD3-APC (145-2c11, Biolegend), F4/80-AF700 (MCA497A700, BioRad), LY6G/6C-APC-Cy7 (RB6-8C5, BD), NK1.1-BV421 (PK136, Biolegend), MHCII-BV711 (M5/114, BD), CD11c-BV785 (N418, Biolegend), CD11b-PE-Cy7 (M1/70, BD), CD45R/B220-V500 (RA3-6B2, BD), CD103-PE (2E7, Invitrogen), CD8-Percp-Cy5.5 (53-6.7, Biolegend) and CD317-BV650 (927, Biolegend).<br><br>CD69-FITC (H1.2F3, Biolegend), CD11c-BV605 (N418, Biolegend), CD4-BV785 (RM4-5, Biolegend), CD11b-PE-DazzleTM594 (M1/70, Biolegend), CD45R-PE-Cy5 (RA3-6B2, Biolegend) and MHC-II-APC (M5/114.15.2, Thermo Scientific), CD3-PE (145-2C11, Thermo Scientific), CD8-PECy7 (53-6.7, Thermo Scientific) and with CD16/CD32 FcγRIII (BD Pharmingen) to block IgG Fc receptors.<br><br>Western Blot Antibodies<br>BMAL1 (14020S, CST), OPA1 (80471, CST), FIS1 (PA5-22142, Thermo fisher Scientific), MFN1 (ab126575, Abcam), MFN2 (9482S, CST), DRP1 (5391S, CST), p-DRP1 (S637) (4867S. CST) and α-Tubulin (3873S, CST) and b-Actin (MAB1501, EMD Millipore) followed by incubation with appropriate Peroxidase-conjugated AffiniPure Goat anti-rabbit IgG (111-0350144, Jackson ImmunoResearch) or Peroxidase-conjugated AffiniPure Goat anti-mouse IgG (115-035-146, Jackson ImmunoResearch).
Validation	Antibodies were validated already on manufacturer's website. Pilot experiments were performed to confirm validation under our experimental conditions.

Eukaryotic cell lines

Policy information about cell lines and Sex and Gender in Research

Cell line source(s)	B16-FLT3L (ATCC B16-F1 RRID: CVCL_IJ12)
Authentication	Cell lines were authenticated by manufacturer (ATCC) and confirmed by microscopy looking at morphology.
Mycoplasma contamination	Cell line was Mycoplasma tested monthly and found to be negative.
Commonly misidentified lines<br>(See ICLAC register)	N/A

Animals and other research organisms

Policy information about studies involving animals; ARRIVE guidelines recommended for reporting animal research, and Sex and Gender in Research

Laboratory animals	All mice used were between 6-10 weeks old at the initiation of experiments. Mice with the gene Bmal1 containing LoxP sites were kindly provided by Christropher Bradfield. Bmal1LoxP/LoxP were crossed with Lyz2Cre mice, which express Cre recombinase under the control of the Lyz2 promoter to produce progeny that have Bmal1 excised in the myeloid lineage. Bmal1LoxP/LoxP::Lyz2Cre (Bmal1myeloid−/−) mice where compared with control Lyz2Cre (Bmal1myeloid+/+). OVA-specific CD4+(OT-II) T-cell receptor-transgenic mice (H-2b)1 for the adoptive transfer of OT-II cells were purchased from Jackson (Strain #:004194) as was PER2::Luciferase (Strain #:006852)
Wild animals	Not used
Reporting on sex	Sex was not reported. Preliminary experiments did not show any differences according to sex.
Field-collected samples	Not used
Ethics oversight	Procedures were approved by the Ethics Committee at Trinity College Dublin and under license from the Ireland Health Products Regulatory Authority (AE19136/P134). All procedures conformed to the Directive 2010/63 EU of the European Parliament.

Note that full information on the approval of the study protocol must also be provided in the manuscript.

Flow Cytometry

Plots

Confirm that:

- [x] The axis labels state the marker and fluorochrome used (e.g. CD4-FITC).
- [x] The axis scales are clearly visible. Include numbers along axes only for bottom left plot of group (a 'group' is an analysis of identical markers).
- [x] All plots are contour plots with outliers or pseudocolor plots.
- [x] A numerical value for number of cells or percentage (with statistics) is provided.

Methodology

Sample preparation	The sample preparation proceedure for each specific flow cytometry experiment is detailed in the Methods section
Instrument	BD Biosciences FACS Canto II cytometer (s/n V96301028) and Cytek Biosciences Aurora spectral analyser s/n is CC376
Software	Canto acquisition software is FACSDiva and Aurora acquisition software is SpectroFlo
Cell population abundance	Cell sorting was not performed.
Gating strategy	Gating strategies are including in supplementary data Figure 1a-b

- [x] Tick this box to confirm that a figure exemplifying the gating strategy is provided in the Supplementary Information.
